# Chromatin state changes during neural development revealed by in vivo cell-type specific profiling

**DOI:** 10.1038/s41467-017-02385-4

**Published:** 2017-12-22

**Authors:** Owen J. Marshall, Andrea H. Brand

**Affiliations:** 1 The Gurdon Institute and Department of Physiology, Development and Neuroscience, University of Cambridge, Tennis Court Road, Cambridge, CB2 1QN UK; 2 Present Address: Menzies Institute for Medical Research, University of Tasmania, 17 Liverpool St, Hobart, TAS 7000 Australia

## Abstract

A key question in developmental biology is how cellular differentiation is controlled during development. While transitions between trithorax-group (TrxG) and polycomb-group (PcG) chromatin states are vital for the differentiation of ES cells to multipotent stem cells, little is known regarding the role of chromatin states during development of the brain. Here we show that large-scale chromatin remodelling occurs during *Drosophila* neural development. We demonstrate that the majority of genes activated during neuronal differentiation are silent in neural stem cells (NSCs) and occupy black chromatin and a TrxG-repressive state. In neurons, almost all key NSC genes are switched off via HP1-mediated repression. PcG-mediated repression does not play a significant role in regulating these genes, but instead regulates lineage-specific transcription factors that control spatial and temporal patterning in the brain. Combined, our data suggest that forms of chromatin other than canonical PcG/TrxG transitions take over key roles during neural development.

## Abstract (web summary)

While transitions between active and repressive chromatin states are essential for differentiation, little is known regarding their role during development of the brain in *Drosophila*. Here, the authors investigate the large scale chromatin remodelling taking place during fly neural development.

## Introduction

Neural development is a complex process that ultimately accounts for all the diverse neuronal subtypes within the adult brain. In *Drosophila*, the majority of neural stem cells (type 1 NSCs) produce ganglion mother cells (or GMCs) that divide once to generate a pair of neurons. Once born, neurons undergo an immature growth phase during which axons are guided to their correct targets; once axon growth is complete and synaptic contacts established, neurons can be considered to be mature. While much is known about neuronal development, an understanding of the key chromatin transitions that drive the process has been lacking. In particular, the genome-wide changes in chromatin state through which large numbers of previously-repressed neuronal genes are activated following differentiation, and through which key NSC genes are repressed, are unknown.

Chromatin has been modelled to different numbers of forms, or states, which can ultimately be reduced to five broad categories^1–5^. Three of these—PcG, TrxG and HP1-asssociated chromatin—have been studied in the context of differentiation of ES cells to neural progenitor cells (NPCs)^6–8^ and PcG modifications have been specifically compared between cultured NPCs and neurons^9, 10^. An overall picture of genes specifying cellular fate being regulated by repressive PcG and active TrxG chromatin has emerged^11–13^, with repressive PcG^10^ and HP1-associated chromatin marks^14, 15^ subsequently spreading over inactive genes in differentiated cells. However, in addition to these three broad categories, two other forms of chromatin have been recently identified in cultured cells: a permissive chromatin state that was not associated with TrxG proteins and encompassed genes involved in metabolism and housekeeping ('Yellow' chromatin in ref. ^4^); and a silent 'Black' (or 'Quiescent' or 'Null') chromatin state that lacked either common histone tail modification marks or known histone-code reader or writer proteins^4^ (cf. state 9 in ref. ^1^ and states 40, 41 and 43 in ref. ^5^). The roles of these two new forms of chromatin during development are unknown, and they have not previously been studied in the context of development or differentiation.

Here we use Targeted DamID to generate cell type-specific chromatin maps of NSCs, GMCs and immature neurons, and mature neurons within the *Drosophila* brain and observe large-scale chromatin remodelling during *Drosophila* neural development. Strikingly, genes involved in establishing NSC or neuronal fate were not repressed by PcG chromatin. Instead, in NSCs they were silent in either a Black chromatin state or a TrxG-repressive state, while in neurons they were repressed via HP1-associated chromatin. We found PcG chromatin to be associated specifically with lineage-specific transcription factors that control the spatial and temporal patterning of the brain.

## Results

### TaDa allows cell type-specific profiling of chromatin states

**Fig. 1:**
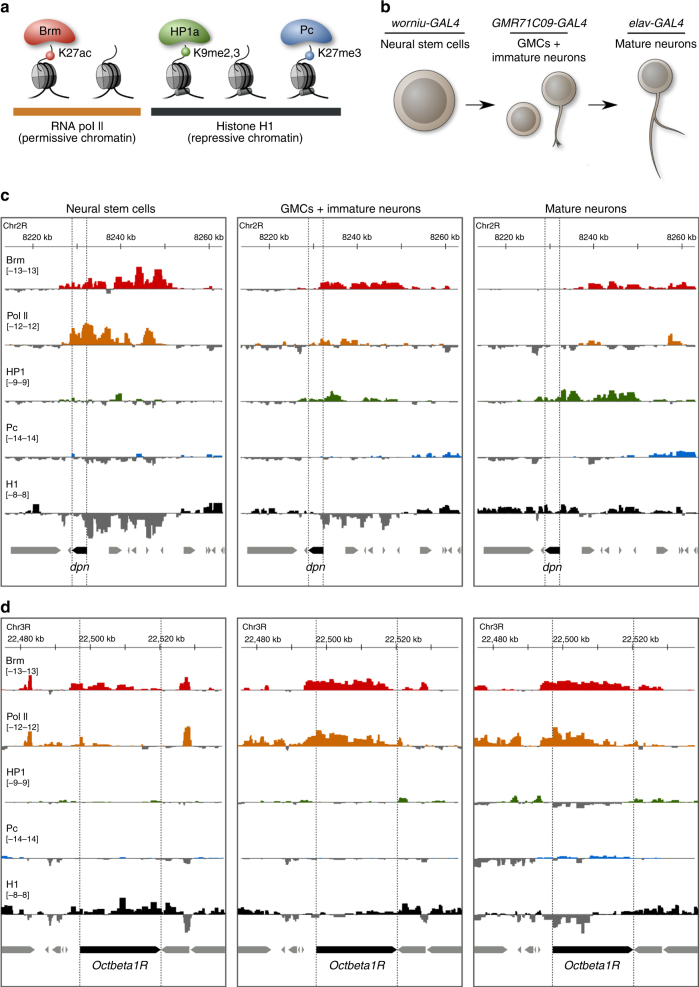
Profiling of in vivo chromatin states during *Drosophila* neural development. **a** Chromatin-associated proteins used in this study. Brm, HP1a and Pc are illustrated alongside the histone H3 modifications that they, respectively, associate with, together with RNA Pol II (open, permissive chromatin) and histone H1 (repressive chromatin). **b** GAL4 driver lines used in this study, and the cell types that they target. **c**, **d** Targeted DamID DNA-binding tracks for the chromatin proteins examined in this study are illustrated for two sample genes. **c** The gene *dpn* (an orthologue of the mammalian *HES* gene family) is rapidly repressed following neuronal differentiation. **d** The octopamine receptor gene *OctbetaR1* is activated following differentiation

To characterise chromatin states during neural development in vivo, we used a technique developed in our lab, Targeted DamID (or TaDa)^16^, which enabled us to determine the cell type-specific DNA-binding profiles of a set of representative chromatin factors: Brahma (a chromatin remodelling protein associated with H2K27ac, and part of a major complex within TrxG chromatin)^17^, HP1 (the H3K9me3 reader, representing HP1-associated heterochromatin)^18, 19^, Polycomb (the H3K27me3 reader, representing PcG chromatin)^20^, histone H1 (the linker histone, enriched in all repressive chromatin)^21^ and the core subunit of RNA Pol II (covering the majority of permissive, actively transcribed chromatin, and allowing transcriptional profiling of each cell type)^16^ (Fig. 1a). The genome-wide binding domains of these proteins were determined in larval NSCs (~15,000 neural stem cells per replicate, driving expression with *worniu-GAL4*), larval GMCs and immature neurons (~60,000 cells per replicate using *GMR71C09-GAL4*) and neurons in newly eclosed adults (~3,000,000 neurons per replicate using *elav-GAL4*) (Fig. 1b). Importantly, Targeted DamID enabled us to observe chromatin changes as they occurred in vivo, in an intact living organism.

The DNA-binding profiles obtained (Fig. 1c, d; Supplementary Fig. 2) illustrate the dynamic nature of chromatin transitions during neural development. As expected, the genes encoding NSC transcription factors such as deadpan (*dpn*) (Fig. 1c; an orthologue of the mammalian *HES* gene family) moved from transcriptionally active chromatin to repressive chromatin, as evidenced by decreased binding of Brahma and Pol II and increased binding of HP1 and histone H1. In contrast, neuronal genes, such as the octopamine receptor gene *Octbeta1R* moved from silent repressive chromatin to transcriptionally active chromatin (Fig. 1d).

**Fig. 2:**
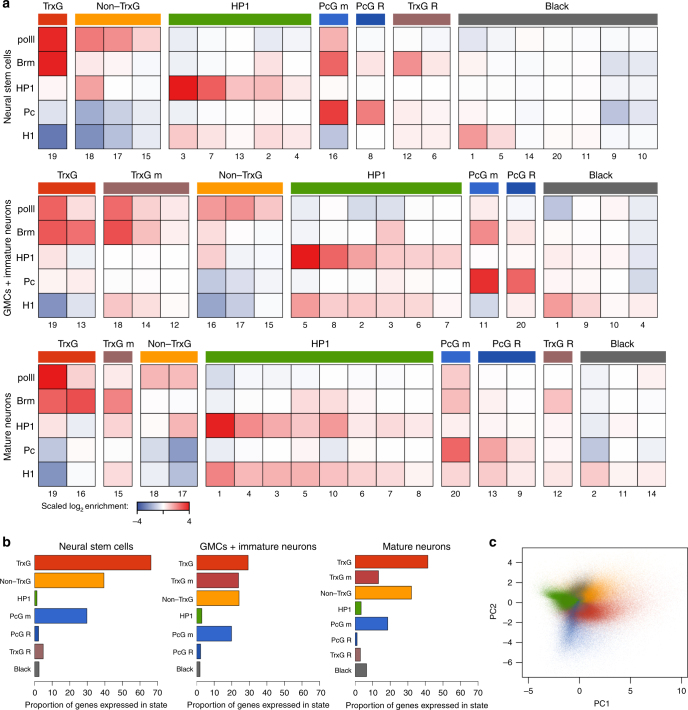
Modelling of chromatin states during neural development. **a** Classification of chromatin marks into 20 states in neural stem cells, GMCs and immature neurons, and mature adult neurons as fitted by hidden markov models. Scaled log_2_ state means are illustrated, with states with similar mean binding grouped into broad chromatin classes. State numbers are arbitrarily assigned during model fitting, and do not correspond between models fitted for the three cell types. **b** Proportion of genes expressed in different chromatin states in each cell type. **c** A PCA plot of all GATC fragments across all cell types indicates clear separation of broad chromatin states

To obtain a genome-wide profile of chromatin states, we fitted hidden markov models (HMMs) to the DNA-binding profiles, fitting separate HMMs to all three data sets (Fig. 2a). Hierarchical cluster analysis of scaled state means (Supplementary Fig. 1b) and state transition probabilities (Supplementary Fig. 1a) allowed fitted states to be compared between cell types and classified into robust categories (Fig. 2a) that separated into distinct clusters through PCA analysis (Fig. 2c). Importantly, we observed five broad chromatin categories in this analysis that were very similar to the five states found in cultured embryonic (Kc167) cells, as previously described by Filion et al.^4^ (Supplementary Fig. 3; Supplementary Table 2). Notably, 60% of Black chromatin coverage was shared between the two data sets, suggesting that we were able to faithfully detect this chromatin state in NSCs. In addition to these five chromatin categories, however, we identified two additional groups of states. Consistent with profiling a population of diverse neuronal lineages, in all three data sets we observed mixed states in which both active and repressive proteins were present. These covered both the TrxG-associated chromatin state ('TrxG mixed') and the PcG-associated chromatin state ('PcG mixed') (Fig. 2a). In both NSCs and mature neurons, we also observed a transcriptionally silent state that contained significant levels of both Brm binding and the linker histone H1, which we termed 'TrxG repressive' (Fig. 2a).

Determining a genome-wide classification of chromatin states in each cell type enabled us to classify genes by their modal chromatin state. Gene expression was calculated from RNA Pol II occupancy^16^, with a clear separation apparent between permissive and repressive states (Fig. 2b). We were then able to investigate the chromatin state transitions occurring between NSCs, GMCs, immature neurons and mature neurons in the adult. A surprisingly high proportion (72.5%) of genes changed their broad chromatin state configuration between the three cell types studied, suggesting a large-scale remodelling of chromatin during neuronal development (Supplementary Table 1).

### Black and TrxG-repressive states silence neuronal genes

**Fig. 3:**
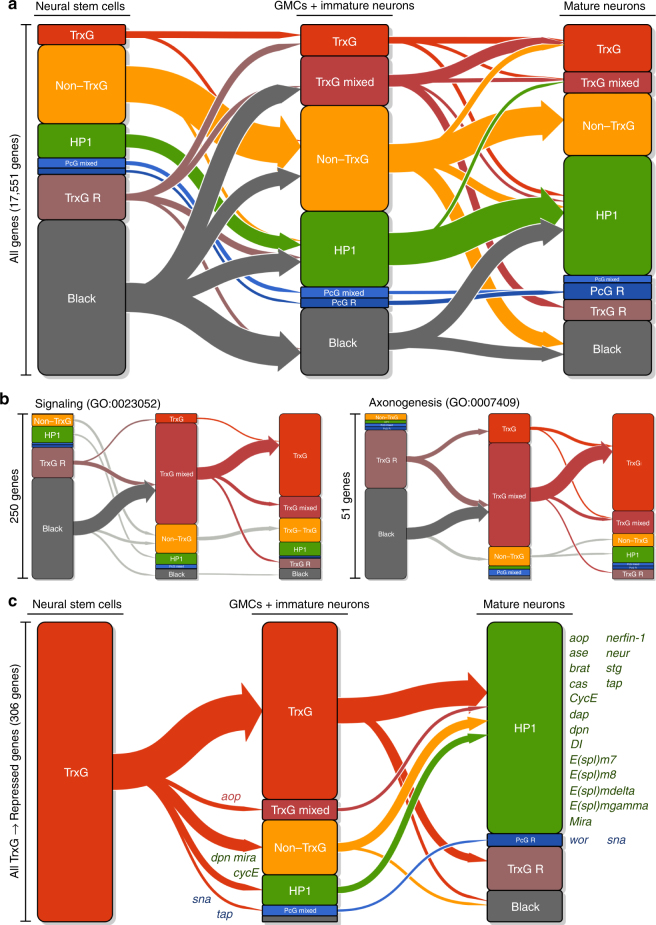
Transitions in chromatin state affect the expression of genes involved in neural development. **a** Chromatin transitions during neuronal development. Proportional bar plots represent the proportion of all annotated genes in each state for each cell type. Arrows indicate the observed chromatin state transitions between cell types, with the width of the arrow proportional to the number of genes undergoing each transition. **b** Novel transitions activate neuronal genes following differentiation. Genes that are not expressed in NSCs and which are turned on in either neuronal cell type are illustrated for the GO term annotations signalling and axonogenesis. **c** Repression of NSC identity genes by HP1-associated chromatin. A transition plot illustrating all genes moving from a TrxG active chromatin state in NSCs to any repressed state in mature neurons is shown. Key genes present in individual transitions are listed alongside the chromatin state

During neuronal differentiation, genes involved in axonogenesis (51 genes, Fig. 3b) and synaptic signalling (250 genes, Fig. 3b) are activated. We found genes for axonal guidance and growth in the TrxG-repressive state in NSCs and then in the TrxG active or TrxG-mixed states in immature neurons (*P* < 10^–6^ for genes involved in axonogenesis, Fig. 3b). Similarly, neurotransmitter and receptor genes (*P* < 10^–11^ for signalling genes, Fig. 3b) and genes for G-protein coupled receptor (GPCR) signalling (*P* < 10^–34^, Supplementary Fig. 5) were in the Black chromatin state and then in a TrxG-mixed state. These genes were activated soon after differentiation, with almost all genes in these categories transitioning from repressive to active or mixed chromatin states as immature neurons (Fig. 3b). Intriguingly, given the importance of TrxG/PcG transitions in controlling the expression of genes in other developmental systems^12^, a role for PcG-regulated chromatin was not immediately apparent for any of these categories of neuronal genes. Indeed, of 1731 genes that were transcriptionally activated as neurons differentiated, only 10 genes transitioned from PcG chromatin.

In addition to the above, we also observed genes that transitioned from the Black chromatin state to the non-TrxG active state (Fig. 3a). Consistent with the previous observation that the non-TrxG active (or 'Yellow') chromatin state corresponds with metabolic and housekeeping genes^4^, this group was enriched for genes regulating metabolic processes (*P* < 10^–7^ for cellular metabolic process genes), as well as protein and vesicle transport (*P* < 10^–4^ for protein transport; *P* = 0.02 for vesicle-mediated transport).

### HP1 chromatin silences NSC identity genes in neurons

As neuronal genes are switched on in neurons, genes that maintain neural stem cell identity are repressed (Fig. 3c). These genes, including *dpn* and *E(spl)-HLHmγ* (orthologues of the mammalian *HES* genes), *ase* (orthologue of the *ASCL* genes) and the cell cycle gene *CycE*, were present in active TrxG-associated chromatin in neural stem cells. Given the importance of TrxG/PcG transitions in other systems, we expected that these genes would also be repressed by PcG-associated chromatin. However, in almost every case neural stem cell fate genes were instead repressed by HP1-associated chromatin (Fig. 3c), with the only exceptions being *wor* (Supplementary Fig. 2a) and *sna* (orthologues of the mammalian *SNAI* gene family). For some genes, the transition from TrxG-associated chromatin to HP1-associated chromatin occurred rapidly, being immediately repressed in immature neurons. Another subset of genes showed a delayed transition, remaining in a permissive TrxG-associated state in immature neurons and only entering a repressive HP1 state in mature neurons. Only the gene encoding *tap* (the neurogenin family orthologue)^22^, and an adjacent lncRNA (*CR45688*) transitioned from TrxG active chromatin to HP1-associated repression through a clear PcG intermediate (Fig. 3c; Supplementary Fig. 2b). The repression of key NSC genes via HP1-associated chromatin was concomitant with a large increase in the proportion of the mapped genome covered by this chromatin state (from 23 Mb in NSCs to 41 Mb in mature neurons; Supplementary Fig. 4; Supplementary Tables 3, 4), consistent with previous observations of an increase in H3K9me2,3 following differentiation of ES cells^14, 15^.

### PcG chromatin controls lineage transcription factors

**Fig. 4:**
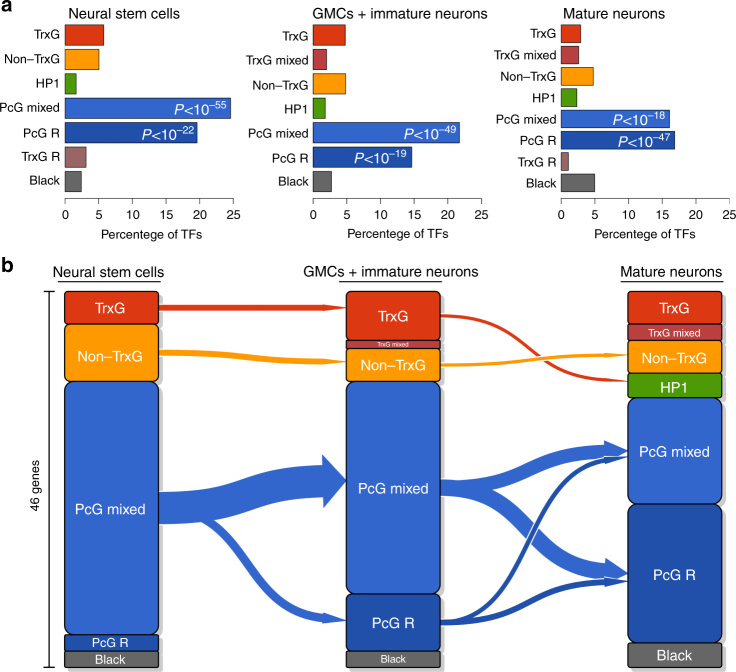
Lineage-specific transcription factors are enriched in PcG-associated chromatin. **a** Enrichment of PcG-associated states for transcription factors. Percentage of transcription factors in each chromatin state are shown, with significant enrichment of transcription factors below *P* < 0.01 denoted (Fisher’s exact test). **b** Transition plot of 46 differentially expressed transcription factors identified from Yang et al.^26^ and expressed with an FDR < 0.01 in at least one of the data sets presented in this study

Given the absence of any major transitions involving PcG chromatin during neuronal differentiation, we sought to understand whether PcG-repressive chromatin played a significant role in neuronal development. In contrast to a previous study of mammalian NPCs differentiated to neurons in culture^10^, we observed little spreading of PcG domains during neuronal differentiation in vivo. Indeed, the overall coverage of PcG-regulated domains remained minor, only increasing from 8.7 Mb in NSCs to 11.5 Mb in mature neurons (Supplementary Fig. 4; Supplementary Tables 3, 4). Importantly, however, our modelled chromatin states distinguished two different forms of PcG-associated chromatin, mixed and repressive, in each cell type. Given the presence of strong RNA Pol II occupancy as well as Brm binding in the mixed PcG state, we reasoned that this state might represent a mix of PcG repression and TrxG activation in cells of different subtypes, rather than classic bivalent chromatin (which is silent and not transcribed). Both states were strongly enriched for transcription factors in each cell type analysed (Fig. 4a; Fisher’s exact test, *P* < 10^–17^ for either PcG-associated state in each cell type studied), and we focused on known transcription factors present within these states for further analysis. Developmentally important transcription factors that are not expressed in NSCs, such as *peb, scr, cad, sob, lab, tin* and *lz*, were present in the PcG-repressive state in NSCs. In contrast, in the PcG-mixed state we found genes encoding transcription factors that regulate the temporal transitions of the NSCs in the larval brain optic lobe *(hth*, *ey, Slp1* and *tll*)^23, 24^ and pattern the optic lobe *(Optix* ^25^ *, Vsx1* ^23^).

The establishment and maintenance of different neuronal subtypes is key to forming a functional nervous system. Our data suggest that, rather than defining the difference in cell type between NSCs and neurons, the role of PcG repression in neural development is to regulate genes involved in establishing and maintaining these specific neuronal lineages. To investigate this hypothesis further, we took advantage of recently published RNA-seq data from three isolated NSC lineages within the *Drosophila* brain at an earlier developmental time point^26^. We identified 46 differentially expressed transcription factors, and plotted the chromatin state transitions occurring at the genes encoding these factors. Of these, 31 (67%; Fisher’s exact test *P* < 10^–31^; Fig. 4b) were present in the PcG-mixed state, the only state that was significantly enriched. Furthermore, these genes remained in either a PcG-mixed or repressed state throughout neural development (Fig. 4b). We suggest that other transcription factors present in the PcG-mixed state (a further 67 transcription factors; Supplementary Table 5) may play a similarly important role in specifying neuronal lineages.

## Discussion

Using Targeted DamID, we have compiled cell type-specific DNA-binding maps of key chromatin proteins in NSCs and neurons, and used these to investigate the chromatin transitions that occur during neural development in the *Drosophila* brain. Our data reveal unexpected roles for chromatin states in this context.

The Black chromatin state—a silent state lacking PcG or HP1a constituents—was identified in cultured embryonic Kc167 *Drosophila* cells^1, 4^, but its role during development was previously unknown. Our data clearly demonstrate the importance of this form of chromatin and the novel TrxG-repressive state during development in vivo, with both states covering the majority of genes that are silent in NSCs but active in neurons. It is not yet known whether Black chromatin is actively repressive and requires chromatin remodelling to become active, or merely passively silent, requiring only the binding of appropriate transcription factors to activate transcription. Enrichment of the linker histone H1 in this state would suggest that chromatin is less accessible. Black chromatin in *Drosophila* and other organisms has been reported also to contain varying levels of the H3K27me2 mark, which may contribute to repression, and which suggested a link to PcG-associated chromatin^27, 28^. Interestingly, however, we observed very few transitions between Black and PcG chromatin: between NSCs and immature neurons, <0.5 Mb of DNA transitioned between Black and PcG states in either direction (Supplementary Table 3) and the PcG-associated states separated strongly from Black chromatin in our models. If Black chromatin does contain H3K27me2 in the brain, there would appear to be little conversion between H3K27me2 and H3K27me3 during neural development.

In addition to many genes that transitioned from Black chromatin in NSCs to an active TrxG state in neurons, we observed a subset of genes that transitioned between Black and non-TrxG active chromatin. These genes were strongly enriched for those encoding metabolic function (in accordance with previous reports of housekeeping and metabolism genes being associated with the non-TrxG active state)^2, 4^ and were activated without the presence of Brm. These transitions may be similar to those recently described in nematodes and *Drosophila* imaginal discs for the activation of developmentally regulated genes without TrxG-associated chromatin marks^29^.

The TrxG-repressive state we describe here is intriguing. This state is enriched for Brm binding in transcriptionally silent chromatin, and covers a large number of neuronal genes in NSCs. PcG-independent repression via the REST complex in ES cells has been reported to be associated with neuronal genes^30^. These genes were found to be present in a poised state that included the TrxG-associated H3K4me3 mark, a situation very similar to the TrxG-repressive state that we observe.

In neural development, we observe little role for PcG repression in controlling broad cell fate, but instead a role in specifying different neuronal lineages. Specific PcG repression/TrxG activation has previously been described for lineage-specific transcription factors within the mushroom body of the brain^31^. A mixed PcG chromatin state has also been previously associated with spatially compartmentalised transcription factors in ChIPseq of whole wing discs^32^, although that study was not cell type-specific. This last study speculated that such a mixed state might be indicative of bivalent chromatin in *Drosophila*.^32^ However, both this study and our own found concomitant occupancy of RNA Pol II on gene bodies in the PcG-mixed state, an observation at odds with silent bivalent chromatin. Combined with a significantly enriched association with lineage-specific transcription factors from existing RNA-seq data and immunofluorescence studies, our data suggest that this chromatin state is a genuine mixture between PcG repression in some lineages and TrxG activation in other lineages. We see no evidence in our data sets of true bivalent chromatin as reported in ES cells (strong binding of both PcG and TrxG components with no transcription).

In conclusion, our data present a picture of neural development that differs from that observed during the differentiation of ES cells in culture. Although lineage-specific transcription factors that generate neuronal diversity are regulated through PcG repression, the majority of genes activated in neurons follow different chromatin transitions. These genes are present in the silent Black chromatin state and a novel TrxG-repressive chromatin state in NSCs and transition to a TrxG permissive chromatin state in immature neurons. Furthermore, we demonstrate that almost all NSC identity and cell cycle genes are repressed in HP1-associated chromatin in neurons, and that this repression occurs concomitantly with a wide-scale accumulation of HP1-associated binding across the genome. Although canonical PcG/TrxG transitions are vital during early development, our data suggest that other forms of chromatin take over important regulatory roles during neural development.

## Methods

### Expression constructs

Details and sequences of all primers used for generating constructs are shown in Supplemental Experimental Procedures. pUAST-attB-LT3 constructs for Brm, Pc, HP1a and H1 were generated by PCR amplifying the coding sequence of each chromatin factor from an embryonic cDNA library with Gibson Assembly adaptor sequences. Primers were designed using PerlPrimer^33^; see Supplementary Table 6 for primer details. The pUAST-attB-LT3-NDam vector^16^ was cut with BglII (NEB), treated with Antarctic phosphatase (NEB), and inserts and cut vector were assembled using the Gibson assembly method^34^. All vectors were sequenced before being used in germline transformation.

### *Drosophila* lines and germline transformation

GAL4 driver lines were *wor-GAL4* ^35^ for neural stem cells, *GMR71C09-GAL4* ^36^ for immature neurons and *w;+;elav-GAL4* ^37^ for mature neurons. All driver lines were crossed with *tub-GAL80* ^*ts*^ on either chromosome 2 or 3 to generate *w;wor-GAL4;tub-GAL80* ^*ts*^, *w;tub-GAL80* ^*ts*^ *;GMR71C09-GAL4*, and *w;tub-GAL80* ^*ts*^ *;elav-GAL4* lines, respectively, for use in Targeted DamID crosses. All drivers were tested for correct cell type-specific expression by crossing to *w;+;UAS-mCD8::GFP* and observing the expression pattern via confocal microscopy under the same conditions as the Targeted DamID sample collections (detailed below).

*UAS-LT3-Dam-Pc* and *UAS-LT3-Dam-HP1a* flies were generated via injection of *w;+;P{CaryP}attP2* embryos with the respective plasmids together with a phiC31 integrase helper plasmid pBS130 as an integrase source; *UAS-LT3-Dam-Brm* and *UAS-LT3-Dam-H1* were generated via injection of *y* ^*1*^ *sc* ^*1*^ *v* ^*1*^ *P{nos-phiC31/int.NLS}X;+;P{CaryP}attP2. UAS-LT3-NDam* and *UAS-LT3-NDam-RpII215* were used as previously described^16^.

### Targeted DamID

Targeted DamID was performed as previously described^16, 38, 39^ with the following modifications. Briefly, for NSC and immature neuronal data sets, flies were allowed to lay on apple juice plates with yeast for 4 h at 25 °C, before transferring plates to 18 °C for 2 days. About 100 larvae from each plate were transferred to food plates and grown at 18 °C for a further 5 days, before shifting to the restrictive temperature of 29 °C. For the NSC data set, expression of the Targeted DamID fusion proteins was induced for 16 h, and 30 brains were dissected per sample per replicate. For the immature data set, expression of the DamID fusion proteins was induced for 24 h and 30 ventral nerve cords were dissected per sample per replicate.

For the mature neuronal data set, flies were allowed to lay at 18 °C for 24 h in food vials. Vials were then kept at 18 °C until eclosion, whereupon the newly eclosed adults were transferred to fresh food vials and grown for a further day at 18 °C. The vials were then shifted to 29 °C for either 24 h (two replicates) or 48 h (two replicates). Following induction, flies were frozen in 14 mL tubes cooled in dry ice. Frozen flies were vortexed and body parts sieved to separate the heads as previously described^40^ using mesh sizes of 710, 425 and 150 µm.

Following tissue isolation, genomic DNA was isolated via DNA micro kits (Qiagen) and digested with *Dpn*I (NEB), before cut DNA was obtained with a PCR purification kit (Qiagen). PCR adaptors were ligated to the cut DNA, before digestion with *Dpn*II (NEB) and PCR amplification (Clontech Advantage cDNA polymerase).

### Next-generation sequencing and data processing

Following the DamID procedure, samples were sonicated in a Bioruptor Plus (Diagenode) to reduce the average DNA fragment size to 300 bp, and DamID adaptors were removed via either overnight *Sau*3AI or *Alw*I digestion. The resulting DNA was purified via magnetic bead clean up using Seramag beads^41^ in 20% (w/v) PEG-8000, and 500 ng of DNA was processed for Illumina Sequencing as previously described^38, 39^. Briefly, DNA fragments were end repaired, A-tailed and ligated with Illumina TruSeq adaptors, cleaned twice with Seramag beads, enriched via six rounds of PCR before a final cleanup with Seramag beads. About 50 bp single-end reads were obtained via either a HiSeq 2500 (Illumina) or HiSeq 1500 (Illumina). Libraries were multiplexed such as to yield at least 20 million mapped reads per sample.

NGS reads in FASTQ format were aligned using the damidseq_pipeline software^39^ using default options for all data sets except for histone H1. For histone H1, samples were normalised using reads per million (RPM) values owing to the low correlation between Dam- and H1-Dam-binding profiles. The resulting gatc.bedgraph data set replicates were compared via Pearson’s correlation and replicates averaged. The mean correlation between replicates was 0.90, with the minimum correlation being 0.81. To allow comparisons between different conditions, averaged data sets were scaled by dividing each data set by its standard deviation. Data sets were visualised using IGV^42^.

### Chromatin state modelling

Processed data from the damidseq_pipeline software in gatc.gff format for the five chromatin proxy proteins was merged, sorted by chromosome and location, and a Gaussian HMM resolved using the RHmm R package (using 2000 random starts and 5–15 iterations per start). Each HMM was fitted for 20 states in order to detect the presence of mixed states within the data; Bayesian information criterion (BIC) was calculated from models fitted from 5 to 20 states for each data set to ensure that the HMMs were not over-fitted. A Viterbi path was then generated from the best fitting model and the original data using the RHmm R package. Each cell type was modelled separately to allow for the possibility that not all chromatin states would be shared between cell types.

Chromatin states were then assigned broad chromatin classes with reference to hclust clustering of state means and HMM state transition probabilities. States with similar mean protein binding and a high shared transition probability were considered to be related and were merged. A simplified Viterbi path was generated from these merged states, and used for subsequent analysis.

To generate the PCA plot in Fig. 2c, the three separate scaled chromatin DamID data sets (NSC, immature neurons, mature neurons) were combined to give a data set of 1.15 × 10^6^ GATC fragments, each with the binding of the five chromatin proteins. PCA was performed using the prcomp function in R, and a plot generated from the first two principle components. Each point was coloured using the assigned chromatin state from the simplified Viterbi paths.

Genes were assigned chromatin states based on the modal state. To allow for the large number of short gene bodies in the *Drosophila* genome and the GATC resolution of the data, the modal state was assigned as the state occupying the plurality of each gene body ±250 bp.

Chromatin state transition graphs were generated in R using the transitionPlot function from the Gmisc R package (http://gforge.se/). Genes encoding transcription factors were determined via a previously published list of all predicted and annotated *Drosophila* transcription factors^43^. All gene ontogeny analysis was carried out using the InterMine perl API^44^. Lists of all genes annotated with a GO term and associated child terms were obtained from the EBI QuickGO site (http://www.ebi.ac.uk/QuickGO/).

All *P*-values listed in the text are Holm–Bonferroni corrected.

### Neuroblast RNA-seq data analysis

Previously published data for RNA-seq of different larval NSC lineages^26^ was obtained from GSE71104. Transcription levels in transcripts per million (TPM) were filtered for replicates, where (mean)/(standard deviation) <1 and replicates were averaged. Genes were assigned as differentially transcribed if the TPM was both >10 in one lineage and <5 in one lineage. All transcription factors within this group, which were also expressed with FDR < 0.01 in at least one data set described in this study, were considered for chromatin state analysis.

### Other bioinformatics analyses

Gene calls from RNA Pol II DamID-seq data were performed on averaged data sets as previously described^16, 39^ using the polii.gene.call R script with default parameters.

All other analyses were performed using R (www.r-project.org).

### Code availability

R scripts for the complete data analysis pipeline are open source and freely available at https://github.com/AHBrand-Lab.

### Data availability

The raw and processed data that support the findings of this study have been deposited in the Gene Expression Omnibus (GEO) repository with accession number GSE77860. Requests for reagents should be addressed to the corresponding author.

## Electronic supplementary material

Supplementary Information
