# Supplementary material for: Chromatin state changes during neural development revealed by in vivo cell-type specific profiling
Source: Nat Commun. 2017 Dec 22;8:2271. doi: 10.1038/s41467-017-02385-4 (PMC5741632; doi:10.1038/s41467-017-02385-4)
Supplement: Supplementary file 1 — Supplementary Information [file 41467_2017_2385_MOESM1_ESM.pdf]

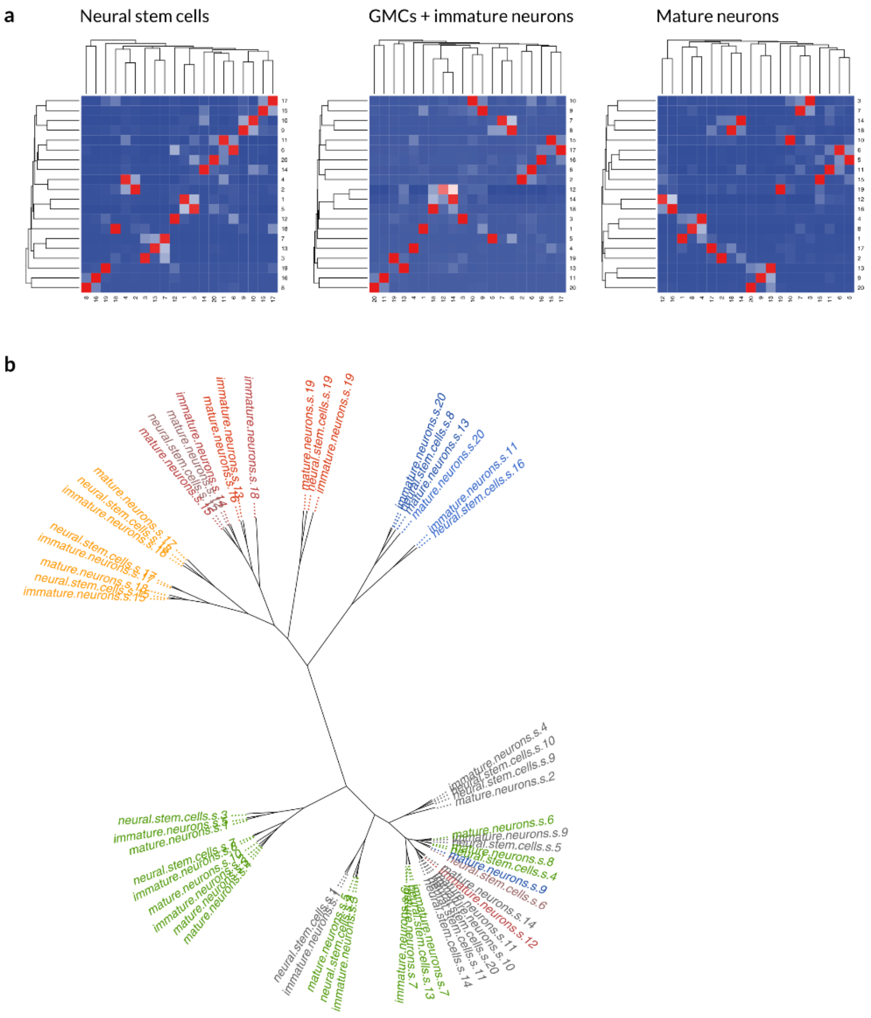

Supplementary Figure 1. Relationships between fitted HMM states in all cell types. (a) The state-state transition probabilities are illustrated as a heatmap for each of neural stem cells, GMCs and immature neurons, and mature neurons. (b) Clustering (hclust) analysis of the scaled mean protein binding of all modelled HMM states across the three cell types studied, represented as an unrooted dendrogram. States are coloured as per the broad chromatin state assignation in Fig. 2. Overall broad chromatin states were assigned based on both transition probabilities and hclust analysis.

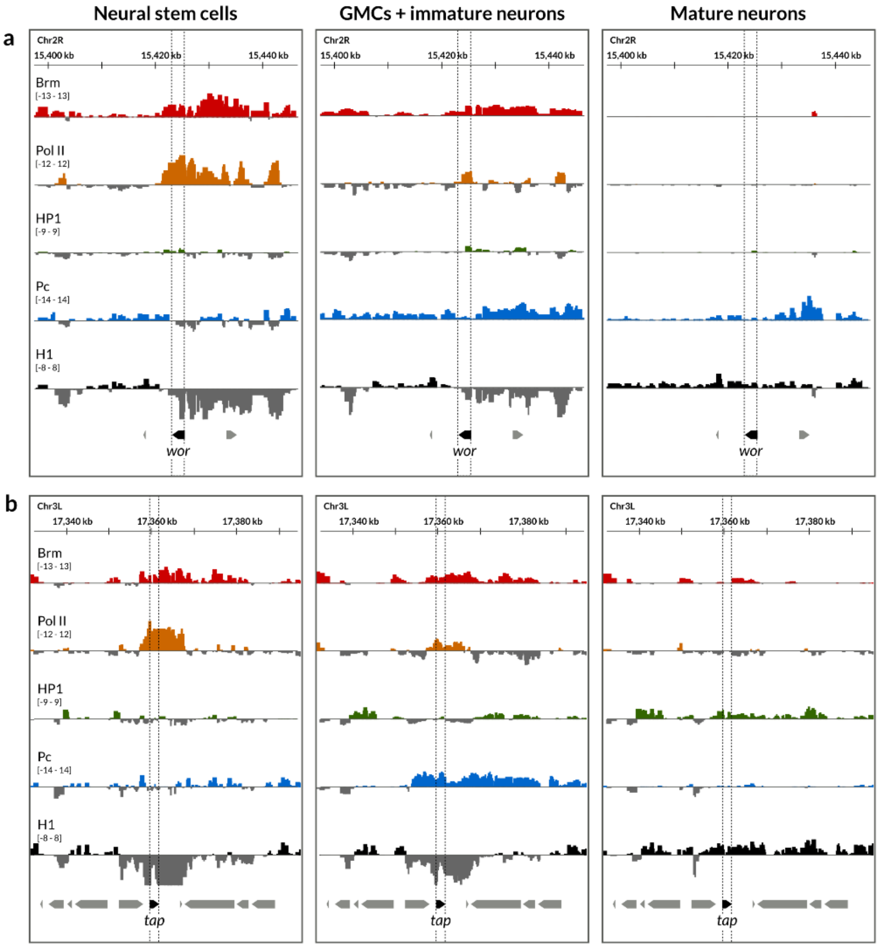

Supplementary Figure 2. Targeted DamID chromatin protein binding plots for two genes within the genome. (a) the Snail-family member transcription factor *worniu*; (b) The neurogenin orthologue *tap*.

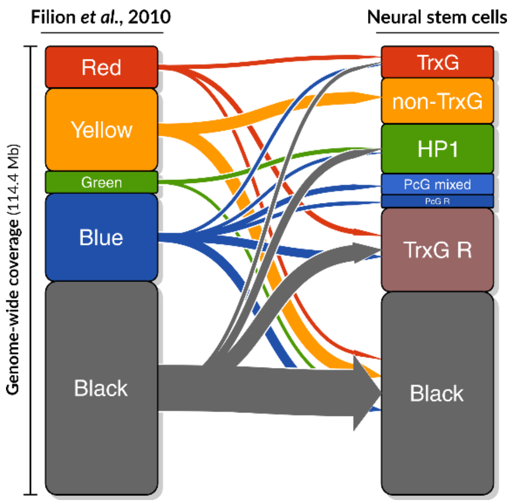

Supplementary Figure 3. Comparison of the chromatin state occupancy from Filion *et al*., 2010 (Kc167 cell line) to this study. The data are presented as a transition for ease of comparison, although embryonic Kc167 cells and larval NSCs are unrelated. Transitions proportional to the amount of mappable chromatin covered are illustrated using the same representation as in Fig. 3.

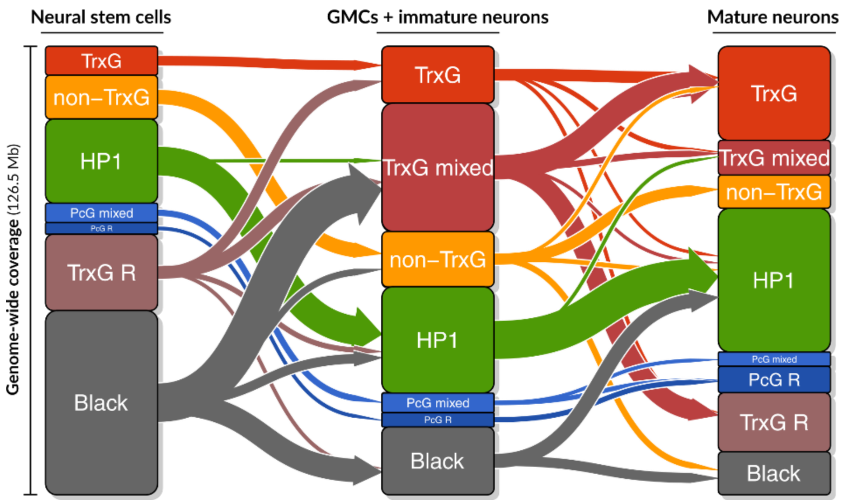

Supplementary Figure 4. Genomic transitions of chromatin states. Transitions proportional to the amount of mappable chromatin covered are illustrated using the same representation as in Fig. 3.

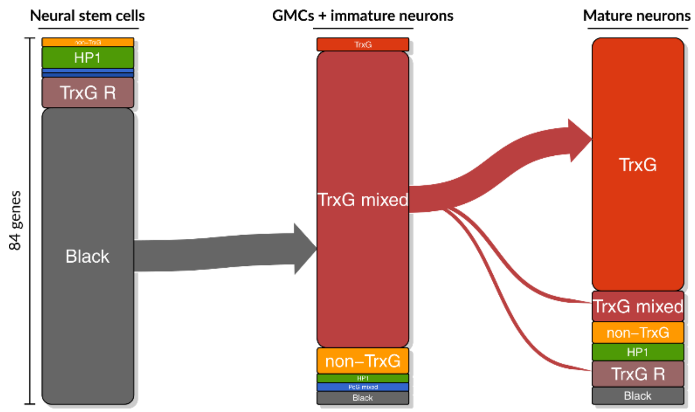

Supplementary Figure 5. Transition plot of genes turned on in the G-protein coupled receptor pathway (GO annotation GO:0007186), illustrated using the same representation as in Fig. 3.

Supplementary Table 1: Number of genes with unchanged chromatin state across all conditions (17283 genes analysed)

Chromatin state	Genes
TrxG permissive	291
non-TrxG permissive	2162
HP1 repressive	993
PcG mixed	140
PcG repressive	162
Black	1006
**Total unchanged**	**4754 (27.5%)**
**Remainder**	**12529 (72.5%)**

Supplementary Table 2: Comparison of genomic chromatin state occupancy between NSCs from this study (columns) and Kc167 cells from Filion *et a*l., 2010 (rows) (Values represent total shared genomic occupancy in Kb)

	TrxG permissive	Non-TrxG permissive	HP1 repressive	PcG mixed	PcG repressive	TrxG repressive	Black
**Red**	2680.03	1094.03	1061.75	332.3	132.76	3237.47	2125.67
**Yellow**	1129.99	8182.06	1787.48	110.95	77.67	1146.7	8748.5
**Green**	50.8	899.08	2685.35	34.59	7.61	99.78	1845.15
**Blue**	1981.56	578.32	1936.47	3532.64	2372.26	5854.77	6216.86
**Black**	2223.92	961.65	5234.42	1385.57	707.14	11079.47	32887.21

Supplementary Table 3: Comparison of genomic chromatin state occupancy between NSCs (rows), and GMCs and immature neurons (columns) (Values represent total shared genomic occupancy in Kb)

	**TrxG permissive**	**TrxG mixed**	**non-TrxG permissive**	**HP1 repressive**	**PcG mixed**	**PcG repressive**	**Black**
**TrxG permissive**	5960.00	467.01	980.56	351.72	299.43	14.37	34.06
**non-TrxG permissive**	827.75	504.27	9087.28	1334.03	28.04	22.85	466.88
**HP1 repressive**	578.70	1826.17	1104.58	18696.68	23.14	46.21	1401.84
**PcG mixed**	889.82	1.61	3.46	33.44	3670.66	826.06	6.72
**PcG repressive**	175.12	34.02	2.27	59.82	963.24	2006.03	52.56
**TrxG repressive**	6534.3	8041.08	421.09	3096.46	458.44	796.13	2075.50
**Black**	1056.35	25275	4006.9	5916.82	55.61	391.07	15033.80

Supplementary Table 4: Comparison of genomic chromatin state occupancy between GMCs and immature neurons (rows), and mature neurons (columns) (Values represent total shared genomic occupancy in Kb)

	**TrxG permissive**	**TrxG mixed**	**non-TrxG permissive**	**HP1 repressive**	**PcG mixed**	**PcG repressive**	**TrxG repressive**	**Black**
**TrxG permissive**	7446.75	2711.04	193.83	1694.70	1092.87	692.27	2067.14	120.71
**TrxG mixed**	15707.57	3790.66	124.24	1819.00	260.55	328.3	12870.15	1317.25
**non-TrxG permissive**	1894.72	279.57	6982.11	2616.97	25.99	29.01	162.26	3624.51
**HP1 repressive**	655.66	1913.07	1154.86	24950.03	96.49	151.47	385.62	676.27
**PcG mixed**	338.37	91.63	38.00	268.77	2007.64	2637.93	91.65	21.49
**PcG repressive**	33.72	28.53	28.10	272.14	440.14	2982.08	146.37	145.42
**Black**	408.53	1006.51	850.04	8948.3	127.65	623.12	911.75	6249.12

Supplementary Table 5: Transcription factors in the PcG-mixed chromatin state in NSCs.

Symbol	Name	Secondary Identifier	DB identifier
Antp	Antennapedia	CG1028	FBgn0260642
B-H2	BarH2	CG5488	FBgn0004854
C15	C15	CG7937	FBgn0004863
CG11085	CG11085	CG11085	FBgn0030408
CG11294	CG11294	CG11294	FBgn0030058
CG12071	CG12071	CG12071	FBgn0039808
CG13287	CG13287	CG13287	FBgn0035643
CG14451	CG14451	CG14451	FBgn0037183
CG15269	CG15269	CG15269	FBgn0028878
CG15696	CG15696	CG15696	FBgn0038833
CG18599	CG18599	CG18599	FBgn0038592
CG31612	CG31612	CG31612	FBgn0051612
CG32105	CG32105	CG32105	FBgn0052105
CG32532	CG32532	CG32532	FBgn0052532
CG34340	CG34340	CG34340	FBgn0085369
CG34367	CG34367	CG34367	FBgn0085396
CG4328	CG4328	CG4328	FBgn0036274
CG43689	CG43689	CG43689	FBgn0263772
CG9650	CG9650	CG9650	FBgn0029939
CR33221	CR33221	CR33221	FBgn0053221
Crg-1	Circadianly Regulated Gene	CG32788	FBgn0021738
Dll	Distal-less	CG3629	FBgn0000157

Symbol	Name	Secondary Identifier	DB identifier
Dr	Drop	CG1897	FBgn0000492
Ets65A	Ets at 65A	CG7018	FBgn0005658
Fer1	48 related 1	CG33323	FBgn0037475
Fer2	48 related 2	CG5952	FBgn0038402
Fer3	48 related 3	CG6913	FBgn0037937
Gsc	Goosecoid	CG2851	FBgn0010323
HGTX	HGTX	CG13475	FBgn0040318
HLH3B	Helix loop helix protein 3B	CG2655	FBgn0011276
HLH4C	Helix loop helix protein 4C	CG3052	FBgn0011277
Hand	Hand	CG18144	FBgn0032209
Hey	Hairy/E(spl)-related with YRPW motif	CG11194	FBgn0027788
Hmx	H6-like-homeobox	CG43748	FBgn0264005
Hr51	Hormone receptor 51	CG16801	FBgn0034012
Lim1	CG11354	CG11354	FBgn0026411
Lim3	Lim3	CG10699	FBgn0002023
Oaz	O/E-associated zinc finger protein	CG42702	FBgn0261613
Optix	Optix	CG18455	FBgn0025360
PHDP	Putative homeodomain protein	CG11182	FBgn0025334
Psc	Posterior sex combs	CG3886	FBgn0005624
Ptx1	Ptx1	CG1447	FBgn0020912
Rfx	Rfx	CG6312	FBgn0020379
Rx	Retinal Homeobox	CG10052	FBgn0020617

Symbol	Name	Secondary Identifier	DB identifier
Six4	Six4	CG3871	FBgn0027364
Sox102F	Sox102F	CG11153	FBgn0039938
Sox21a	Sox21a	CG7345	FBgn0036411
Sox21b	Sox21b	CG32139	FBgn0042630
Sp1	Sp1	CG1343	FBgn0020378
Su(z)2	Suppressor of zeste 2	CG3905	FBgn0265623
TfAP-2	Transcription factor AP-2	CG7807	FBgn0261953
Vsx1	Visual system homeobox 1 ortholog	CG4136	FBgn0263511
Vsx2	Visual system homeobox 2 ortholog	CG33980	FBgn0263512
abd-A	abdominal A	CG10325	FBgn0000014
ap	apterous	CG8376	FBgn0267978
ato	atonal	CG7508	FBgn0010433
bap	bagpipe	CG7902	FBgn0004862
bi	bifid	CG3578	FBgn0000179
btd	buttonhead	CG12653	FBgn0000233
croc	crocodile	CG5069	FBgn0014143
dac	dachshund	CG4952	FBgn0005677
disco	disconnected	CG9908	FBgn0000459
dmrt93B	doublesex-Mab related 93B	CG5737	FBgn0038851
dmrt99B	doublesex-Mab related 99B	CG15504	FBgn0039683
drm	drumstick	CG10016	FBgn0024244
ems	empty spiracles	CG2988	FBgn0000576

Symbol	Name	Secondary Identifier	DB identifier
en	engrailed	CG9015	FBgn0000577
erm	earmuff	CG31670	FBgn0031375
esg	escargot	CG3758	FBgn0001981
eve	even skipped	CG2328	FBgn0000606
exex	extra-extra	CG8254	FBgn0041156
ey	eyeless	CG1464	FBgn0005558
fd102C	forkhead domain 102C	CG11152	FBgn0039937
fd3F	forkhead domain 3F	CG44123	FBgn0264954
fd59A	forkhead domain 59A	CG3668	FBgn0004896
fd96Ca	forkhead domain 96Ca	CG11921	FBgn0004897
fd96Cb	forkhead domain 96Cb	CG11922	FBgn0004898
fkh	fork head	CG10002	FBgn0000659
ftz	fushi tarazu	CG2047	FBgn0001077
gcm	glial cells missing	CG12245	FBgn0014179
gcm2	gcm2	CG3858	FBgn0019809
grn	grain	CG9656	FBgn0001138
gsb	gooseberry	CG3388	FBgn0001148
gsb-n	gooseberry-neuro	CG2692	FBgn0001147
gt	giant	CG7952	FBgn0001150
ham	hamlet	CG31753	FBgn0045852
hbn	homeobrain	CG33152	FBgn0008636
hkb	huckebein	CG9768	FBgn0261434

Symbol	Name	Secondary Identifier	DB identifier
hth	homothorax	CG17117	FBgn0001235
ind	intermediate neuroblasts defective	CG11551	FBgn0025776
kn	knot	CG10197	FBgn0001319
kni	knirps	CG4717	FBgn0001320
lov	jim lovell	CG16778	FBgn0266129
mid	midline	CG6634	FBgn0261963
mirr	mirror	CG10601	FBgn0014343
oc	ocelliless	CG12154	FBgn0004102
opa	odd paired	CG1133	FBgn0003002
otp	orthopedia	CG10036	FBgn0015524
pan	pangolin	CG34403	FBgn0085432
ph-p	polyhomeotic proximal	CG18412	FBgn0004861
pnt	pointed	CG17077	FBgn0003118
repo	reversed polarity	CG31240	FBgn0011701
retn	retained	CG5403	FBgn0004795
ro	rough	CG6348	FBgn0003267
run	runt	CG1849	FBgn0003300
salm	spalt major	CG6464	FBgn0261648
sens-2	senseless-2	CG31632	FBgn0051632
sim	single-minded	CG7771	FBgn0004666
slou	slouch	CG6534	FBgn0002941
slp1	sloppy paired 1	CG16738	FBgn0003430

Symbol	Name	Secondary Identifier	DB identifier
slp2	sloppy paired 2	CG2939	FBgn0004567
so	sine oculis	CG11121	FBgn0003460
sv	shaven	CG11049	FBgn0005561
svp	seven up	CG11502	FBgn0003651
tll	tailless	CG1378	FBgn0003720
toy	twin of eyeless	CG11186	FBgn0019650
tsh	teashirt	CG1374	FBgn0003866
tup	tailup	CG10619	FBgn0003896
unc-4	unc-4	CG6269	FBgn0024184
unpg	unplugged	CG1650	FBgn0015561
vnd	ventral nervous system defective	CG6172	FBgn0261930
vvl	ventral veins lacking	CG10037	FBgn0086680
zfh2	Zn finger homeodomain 2	CG1449	FBgn0004607

Supplementary Methods

Cloning primers for creating Targeted DamID chromatin constructs

Supplementary Table 6: Primer sequences used for generating chromatin constructs. Lowercase letters represent overlap sequences for Gibson Assembly into the pUAST-attB-LT3-NDam vector.

Primer name	Sequence
Pc-Fw	acagaaactcatctctgaagaggatctgcgAGATCTAATGACTGGTCGAGGCAAGG
Pc-Rev	cctctagaggtaccctcgagccgcggccgcaCTCAAGCTACTGGCGACGA

Primer name	Sequence
H1-Fw	acagaaactcatctctgaagaggatctgcgAGATCTGATGTCTGATTCTGCAGTTGC
H1-Rev	cctctagaggtaccctcgagccgcggccgcaTGCGAACATGTACCAAATACTG
HP1a-Fw	acagaaactcatctctgaagaggatctgcgAGATCTAATGGGCAAGAAAATCGACAAC
HP1a-Rev	cctctagaggtaccctcgagccgcggccgcaGCTTTCGATGATCCAACTGTTT
Brm-Fw	acagaaactcatctctgaagaggatctgcgAGATCTCAATATGGCCTCGCCCTCTC
Brm-Rev	cctctagaggtaccctcgagccgcggccgcaGGTAGCTAGTCCATGTCATCGT
